# Nonlinear collision between propagating waves in mouse somatosensory cortex

**DOI:** 10.1038/s41598-021-99057-7

**Published:** 2021-10-04

**Authors:** M. Di Volo, I. Férézou

**Affiliations:** 1 Laboratoire de Physique Théorique et Modélisation, CY Cergy Paris Université, 95302 Cergy-Pontoise Cedex, France; 2 Université Paris-Saclay, CNRS, Institut des Neurosciences Paris-Saclay, Gif-sur-Yvette, France

**Keywords:** Computational neuroscience, Network models

## Abstract

How does cellular organization shape the spatio-temporal patterns of activity in the cortex while processing sensory information? After measuring the propagation of activity in the mouse primary somatosensory cortex (S1) in response to single whisker deflections with Voltage Sensitive Dye (VSD) imaging, we developed a two dimensional model of S1. We designed an inference method to reconstruct model parameters from VSD data, revealing that a spatially heterogeneous organization of synaptic strengths between pyramidal neurons in S1 is likely to be responsible for the heterogeneous spatio-temporal patterns of activity measured experimentally. The model shows that, for strong enough excitatory cortical interactions, whisker deflections generate a propagating wave in S1. Finally, we report that two consecutive stimuli activating different spatial locations in S1 generate two waves which collide sub-linearly, giving rise to a suppressive wave. In the inferred model, the suppressive wave is explained by a lower sensitivity to external perturbations of neural networks during activated states.

## Introduction

The 4th layer of the rodent primary somatosensory (S1) cortex has a remarkable cellular organization, with obvious structures, named “barrels”, laid out with a specific topology corresponding to the whiskers on the snout of the animal ^1^. A precise tactile stimulation of a specific whisker on the snout of the animal activates primarily the corresponding barrel in S1 ^2–4^. Because of its structure, considered as a manifestation of the columnar functional organization of the cerebral cortex, S1 has been largely studied in order to understand the way cortex processes tactile sensory information. It constitutes an ideal framework where to study the emergence of complex spatio-temporal patterns of activity in response to discrete sensory stimulations. Indeed, while a temporally defined individual whisker stimulation activates at first its corresponding barrel in S1, the activity rapidly propagates to the neighboring barrel-related columns, activating in a few tens of milliseconds almost the whole S1. Such spatio-temporal propagation can be measured in S1 using VSD imaging^5–7^, revealing that the simple stimulation of one whisker generates a complex representation across space and time in S1. In this work we investigate, by combining VSD imaging (VSDi) and computational modeling, how spatially distributed cellular connectivity in S1 regulates the emergence of anisotropic propagating waves in S1, meaning waves of activity whose intensity is not the same in all directions. Moreover, we characterize S1 response to more complex stimuli (more precisely, successive deflection of two distinct whiskers), where the collision between propagating waves of activity (originating at different locations) determines the way tactile stimulations are represented in the cortex.

Measuring S1 activity in anesthetized mice in response to tactile stimulations with VSD, we developed a biologically realistic two dimensional cortical model of S1 composed by interconnected networks of excitatory and inhibitory neurons. Indeed, mean field models capture the level of integration of hundreds of neurons, as it is measured in VSDi data^8^. The model we propose employs a mean field approach to mimic the activity of populations of excitatory and inhibitory neurons. This approach has the advantage to describe the dynamics of a population of neurons with a low dimensional model ^9,10^ but, at the same time, to be capable to encode biologically realistic ingredients (indeed we have recently shown that it is applicable to any spiking neuronal model ^11^). A previous version of this model (one dimensional in space) has been employed to study the emergence of propagating waves in the primary visual cortex of the awake monkey ^12,13^. For a quantitative comparison with VSD images, we have here developed a two dimensional model that also includes spike frequency adaptation observed in excitatory regular spiking neurons in the cortex ^14,15^. Moreover, we have employed the model to predict the activity in S1 in response to the successive stimulation of two different but spatially close whiskers. The stimulation of one of the two whiskers produces a propagating wave that in few milliseconds reaches the spatial location of the representation of the other whisker. As a result, if the two stimuli are presented at a close time interval the two propagating waves collide. The model predicts a sublinear interaction between propagating waves in S1 due to lower sensitivity to external stimuli of cortical populations during high levels of activity. This prediction has been directly tested in experiments where we have indeed measured a suppressive wave (sublinear interaction) that propagates from the second stimuli location towards the first one. Nonlinearities in cortical activity have been reported in the context of visual illusion^16^ and, most recently, a similar suppressive wave as the one we observed here has been reported in the monkey primary visual cortex, where two consecutive and spatially close visual stimuli cause the collision of propagating waves with a suppressive interaction ^13^. Our results reveal that this phenomenon is generally present in different animal models and brain regions.

## Results

### Anisotropic structure of propagating waves in S1

**Figure 1:**
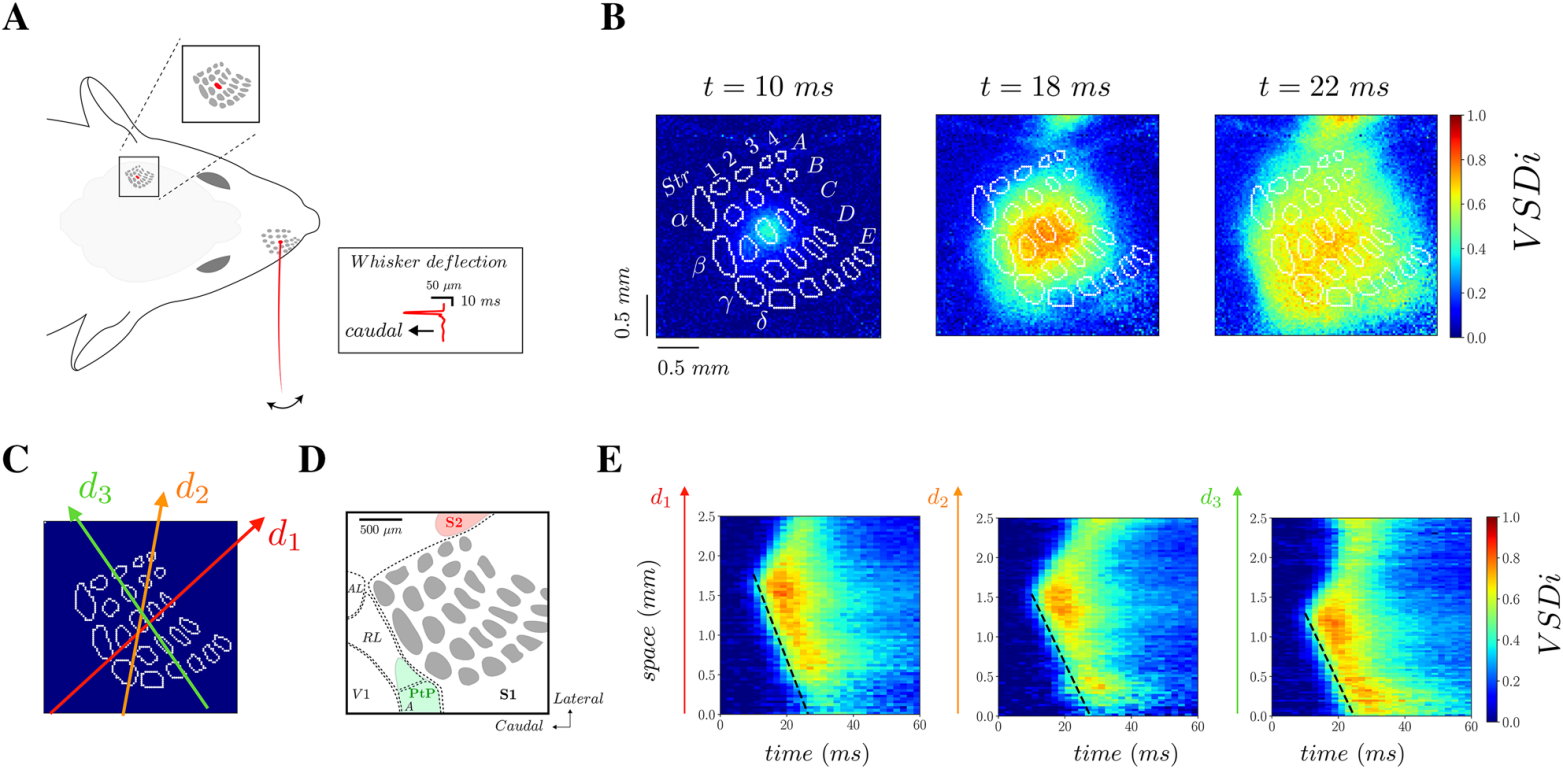
Experimental protocol and anisotropic propagating waves in S1. **(A)** Experimental setup where the S1 is imaged at 500 images per second while the C2 whisker is stimulated with a piezoelectric actuator (2.7° displacement, with a 2-ms rising ramp, 2-ms plateau, and 2-ms fall). (**B)** Color plots show images at different time frames after the beginning of the C2 whisker deflection (t = 0 ms). Intensity of the VSD signal is color-coded and normalized to the maximum intensity in space and time (average of n = 40 trials). The layer 4 barrel map reconstructed from a post hoc cytochrome oxidase staining is shown as overlaid white dots. (**C)** The three arrows (d_1_. red, d_2_. orange and d_3_. green) indicate different spatial directions that we employ for a spatio-temporal visualization of VSD activity (linescan plots on panel **E**). We have estimated, in each direction, the velocity of propagation through a fitting procedure based on the rise of activity in time at each spatial location. The fits (v_c_ = 0.1 + /− 0.01 m/s for the three directions) are reported as dashed black lines on the plots. (**D)** Typical field of view covering the whiskers representation area within S1 (barrels are shown in grey), with the approximated locations of the secondary somatosensory area (S2, as reported from^20^) and the posterior part of the parietal cortex (PtP), as reported from^21^ (*V1* primary visual area, *AL* anterolateral area, *RL* rostrolateral area, *A* anterior area). (**D)** Linescan plots in the directions shown in panel (**C)**.

We measured the spatio-temporal activity of the mouse S1 at 500 Hz by means of VSD imaging ^17,18^. As previously reported ^7^ a small deflection of the right C2 whisker (see “Methods”), evokes a response emerging in less than ten milliseconds at the corresponding barrel-related column location in the left S1. The signal then propagates in space during the following milliseconds, as it can be observed by looking at the pattern of activity at different time frames (Fig. 1A,B). Note that the outlines of S1 barrels could be overlaid on the functional images by means of post-hoc reconstruction of the barrel map from cytochrome oxidase stained sections, as previously described ^19^. This propagation is not uniform, as further revealed by linescan plots (time on x axis and space on y axis) of the VSD signal from lines drawn in different directions (Fig. 1C). Indeed, according to their orientation, the linescan plots show different spatio-temporal patterns of activity that are not symmetric with respect to the initial activation site. Dashed lines in Fig. 1C show the estimation of propagation velocity in different directions. The velocity is estimated by calculating the spatial location when the VSD signal exceeds a fixed threshold. In contrast to the intensity of the VSD signal, we found that the velocity of propagation (v_c = 0.1 mm/ms) is approximately the same in all directions. For a gross identification of the regions surrounding the barrel field in our field of view, we took advantage of our recently reported functional imaging data in the secondary somatosensory cortex (S2)^20^, and a detailed histological study of the posterior parietal cortex^21^ (Fig. 1D). In next sections we develop a mean field model of S1 to study how the emergent anisotropic structure of VSD activity depends on a heterogeneous distribution of neuronal connectivity in space.

### A computational model for propagating waves in sensory cortex

**Figure 2:**
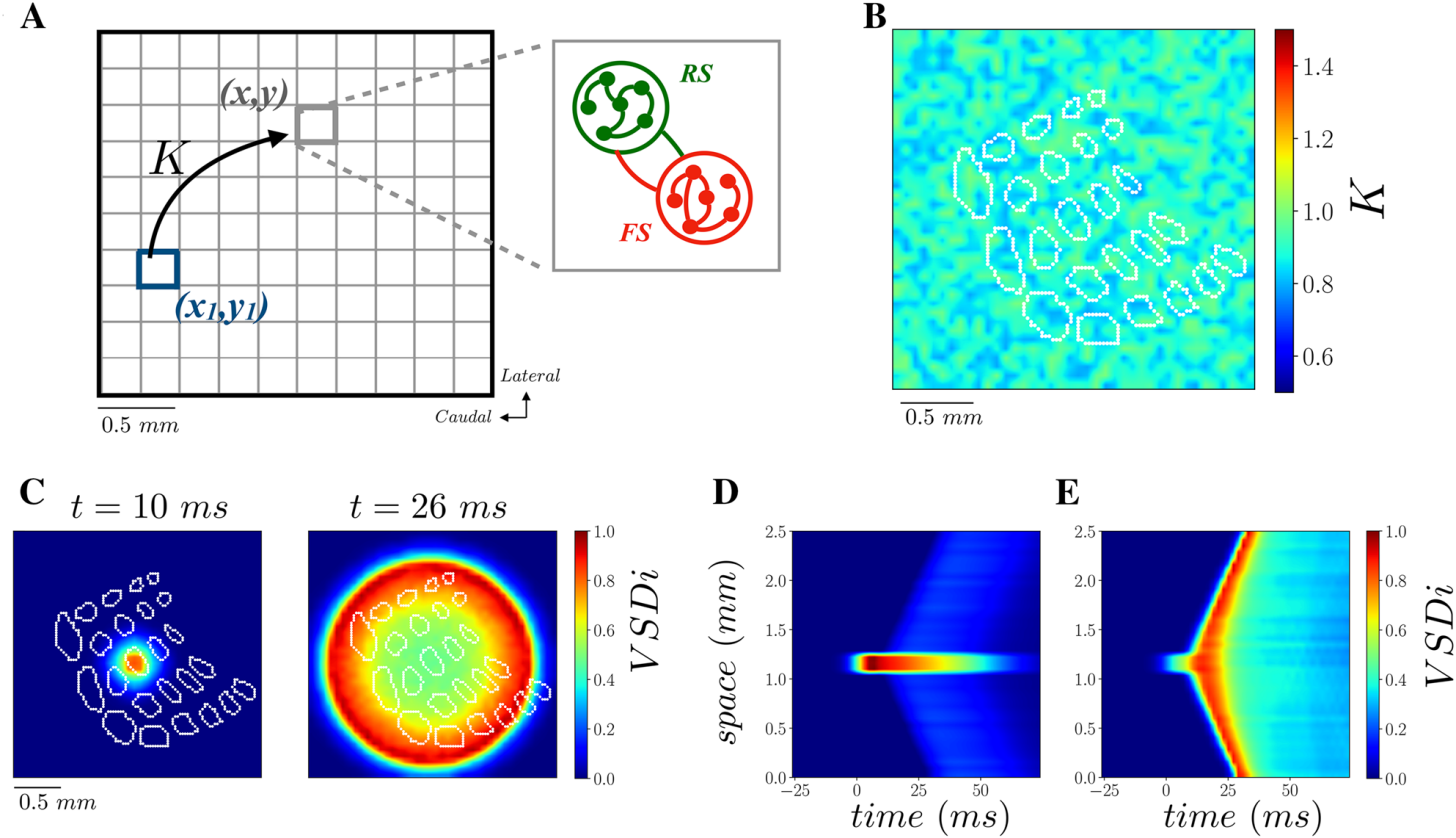
Computational model of S1. **(A)** A two dimensional lattice is considered to model activity in S1. Each point at a location (x,y) is modelled as a population of adaptive excitatory Regular Spiking (RS) and non-adaptive Fast Spiking (FS) inhibitory neurons. (**B)** The strength of the excitatory incoming synapses (quantal conductance) Q_E_ = Q^0^_E_* K(x,y) is homogeneously distributed around its average Q^0^_E_ (K is extracted from a flat distribution with average < K >  = 1 and width ΔK = 0.4). The color plot shows the values of K in the lattice for this flat and homogeneous distribution. (**C)** Activity pattern with the distribution as in (**B)** a pulse of stimulation in C2 at time t = 0 ms evokes an isotropic propagating wave. (**D)** Spatio-temporal activity along the vertical direction passing by C2, same parameters as in (**C)**. (**E)** Spatio-temporal activity along the vertical direction passing by C2 with a uniform *K*, as in **(D)**, but with a lower average < *K* >  = 0.4 (instead of < *K* >  = 1 in panel **B–D**).

We developed a two dimensional computational model of S1 to be compared with imaging data, by extending a mean-field model recently employed to reproduce accurately, in one dimension, VSD signals evoked by visual stimuli in the primary visual cortex of awake primates ^12,13^. We thus performed a discretization in space where each node of the resulting lattice is composed by two populations of interacting neurons, excitatory regular-spiking (RS) neurons, and inhibitory fast-spiking (FS) neurons (Fig. 2A). Excitatory RS neurons, in contrast to inhibitory FS cells, are characterized by spike frequency adaptation, in agreement with experimental observations^14,22^. The mean field model gives access to the average membrane potential of the whole population, from which we estimate VSD intensity (see methods for details). A remarkable propriety of propagating waves in S1 is their spatial extent. Indeed, the stimulation, as well as the resulting first activation in S1, is extremely confined spatially (Fig. 1A). Nevertheless, the wave propagates far away from the activation site at speed v_c_ with high intensity. The intensity of the signal in space is approximately the same of the intensity at the source (see Fig. 1C, direction d_3_). A fundamental ingredient for stimulus-evoked propagating waves at the mesoscopic scale is the conduction velocity of the axons in superficial layers ^23^. Moreover, stimulus-evoked activity propagates with sustained intensity in bistable systems where the inputs traveling along axons trigger, with a space-dependent time delay, jumps from a low to a higher activity state ^24^. We employ, for each population of neurons (RS and FS), a recently developed mean field model with spike frequency adaptation ^15^. Spike frequency adaptation can give rise to a bidirectional switch from low to high activity states ^25–27^. Indeed, a pulse of external excitatory drive to the network can provoke the jump from a low to a high activity state. Because spike frequency adaptation builds up with increasing excitatory neurons activity, the activity then subsequently decreases. The hyperpolarization of excitatory neurons leads then to a decrease of activity and a jump back to the low activity state ^15^. When arranging several of these populations in a homogeneous space, a local input can potentially trigger a chain of jumps to high activity states that spreads in space. Nevertheless, the intensity of such activity is crucially shaped by heterogeneities in the system. Indeed, in a large variety of fields where propagating waves have been under scrutiny, e.g. experiments on the Belousov–Zhabotinsky reaction (BZR) ^28^ or on cardiac tissue ^29^, it has been shown that spatial heterogeneity can strongly affect the emergent structure of their spatiotemporal-dynamics. In our model, a crucial role is played by the spatial distribution of coupling strength between subpopulations of neurons. In this work we make the hypothesis that the coupling strength from a node (x_1_,y_1_) to another node (x,y) in space (Fig. 2A), namely ${G}^\frac{E}{I}(x,y;{x}_{1},{y}_{1})$, is exponentially decreasing with the Euclidean distance $d$. Moreover, we assume this coupling to be proportional to the strength of incoming synapses in (x,y), say ${K}^\frac{E}{I}(x,y)$ (see methods). The higher ${K}^\frac{E}{I}(x,y)$, the stronger is the excitatory/inhibitory (E/I) input incoming in one neuron in (x,y). In our mean field formalism, ${K}^{E}(x,y)$ can be interpreted as the average amount of incoming excitatory connections in (x,y), or as the average strength of excitatory synaptic conductances for one neuron in (x,y). Notice that FS neurons do not have spike frequency adaptation and, as a result, they have a higher gain with respect to RS neurons ^14^. In the case where ${K}^{E}(x,y)$=${K}^{I}(x,y)=K$ is extracted from a spatially uniform distribution around 1 (see Fig. 2B), we observe that a pulse of excitatory activity in the location of C2 activates a propagating wave that spreads uniformly in space with a speed equal to the axon conduction velocity in the model v = v_c = 0.1 m/s (Fig. 2C,D). If we consider instead a lower excitatory coupling ${K}^{E}(x,y)$ (but still uniform), we observe that the activity that is triggered by the external stimuli in C2 does not propagate with the same intensity in space (Fig. 2E). As a result, the emergence and the spatial structure of the propagating wave are crucially shaped by the average value and the spatial structure of ${K}^{E}(x,y)$. In the next section we show how it is possible to employ the model in an inverse way, in order to estimate ${K}^{E}(x,y)$ directly from VSD recordings.

### Inferring spatial heterogeneity from propagating waves in sensory cortex

We develop here a method to infer the coupling distribution ${K}^{E}(x,y)$ from the VSD measure of activity in S1. From the measured spatio-temporal VSD pattern, we reconstructed ${K}^{E}(x,y)$ through a minimization procedure. Starting from a uniform ${K}^{E}(x,y)$ (we have also used different initial conditions) we search for the distribution ${K}^{E}(x,y)$ such that the spatio-temporal activity measured experimentally matches the one predicted by the model. We start with a flat uniform distribution (as in Fig. 2B) and we modify ${K}^{E}(x,y)$ until the relative error between known and predicted VSD activity (integrated in space and time) is smaller than 10%. More details on the procedure are found in the method section. The inversion procedure could potentially be applied also to other parameters such as ${K}^{I}(x,y)$. Nevertheless, we focus here on the excitatory coupling keeping uniform ${K}^{I}(x,y)$ because the response of a population of neurons depends mainly on the ratio between excitation and inhibition ^15^. In this view, modifying ${K}^{E}(x,y)$ and freezing ${K}^{I}(x,y)$ can be seen as modifying the ratio between excitatory and inhibitory coupling. This is why in the following we will focus on ${K}^{E}(x,y)$, calling it $K(x,y)$ for simplicity. Nevertheless, in future work we plan to extend the method to reconstruct both the excitatory and inhibitory couplings (see discussion section).

**Figure 3:**
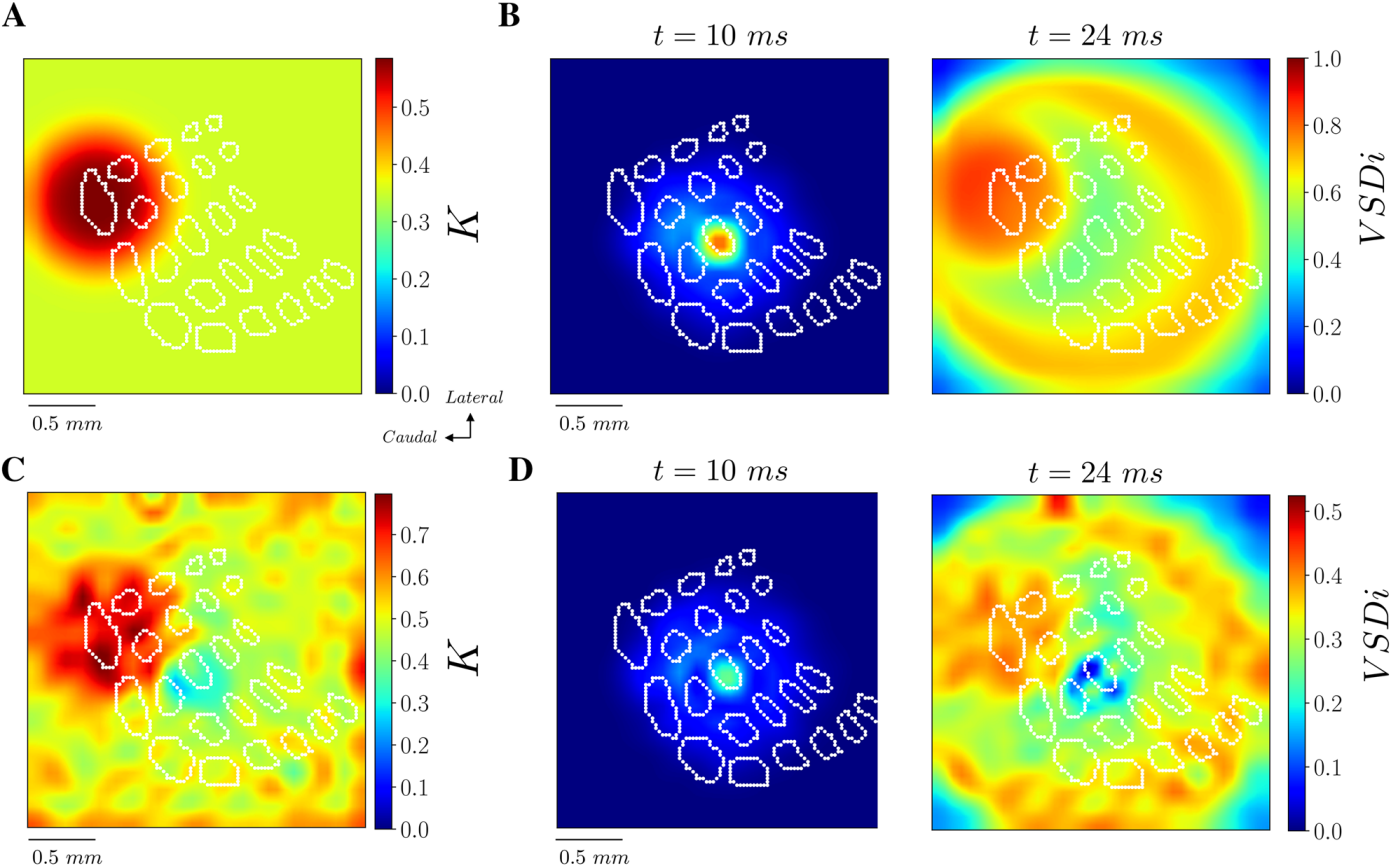
Spatial couplings inferred from synthetic VSD imaging data. **(A)** Spatial distribution of couplings K. (**B)** Propagating wave emerging in response to a C2 whisker stimulus (at t = 0 ms) in the computational model with couplings as in (**A)**. (**C)** Reconstructed distribution of coupling K with the inversion procedure applied to the VSD activity from (**B)**. (**D)** Propagating wave with the reconstructed distribution of coupling K as in (**C)**.

First of all we check the validity of the procedure by considering a well-known $K(x,y)$ with a non-uniform structure (Fig. 3A). We then generate from numerical simulations of the model a synthetic version of VSD data where a propagating wave is originated by a pulse of stimulation in C2 (see methods). These synthetic VSD data are characterized by a spatially non-uniform propagating wave (Fig. 3B). We then employ the inversion procedure from the knowledge of only the synthetic spatio-temporal VSD signal numerically simulated (the spatio-temporal patterns as in Fig. 3B). We observe that the inversion procedure converges to a $K(x,y)$ very similar to the real one (compare Fig. 3C and Fig. 3A). Moreover, the wave predicted by the model with this reconstructed $K(x,y)$ is indeed close to the real one (see Fig. 3D vs B).

**Figure 4:**
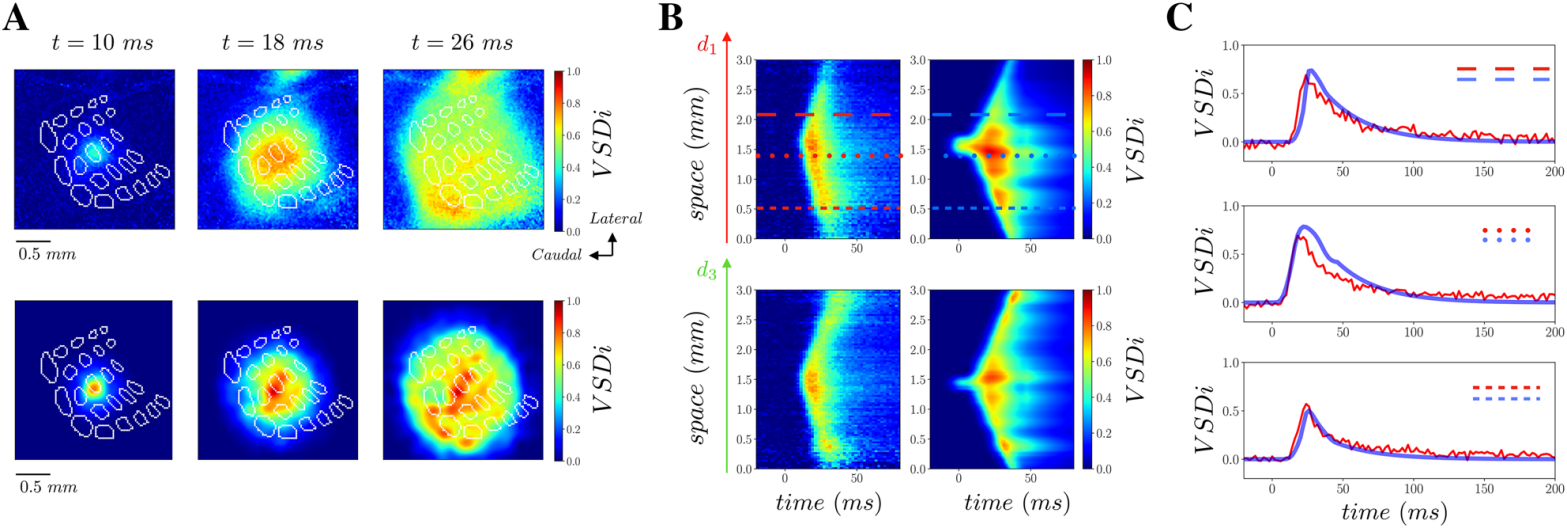
Inference from experimental data. **(A)** Time frames of VSD activity evoked by a C2 whisker stimulus (at t = 0 ms) from data (top row) and from the model with reconstructed couplings (bottom row). (**B**) Spatio-temporal linescan plots computed from data (left) and the model (right) for two different spatial segments (see Fig. 1C). (**C**) Time traces of VSD activity (normalized to its maximum in space and in time) in time at the three spatial location marked as dashed, dotted and densely dashed lines marked in **(B)**. Red (blue) solid line are from experimental data (computational model).

**Figure 5:**
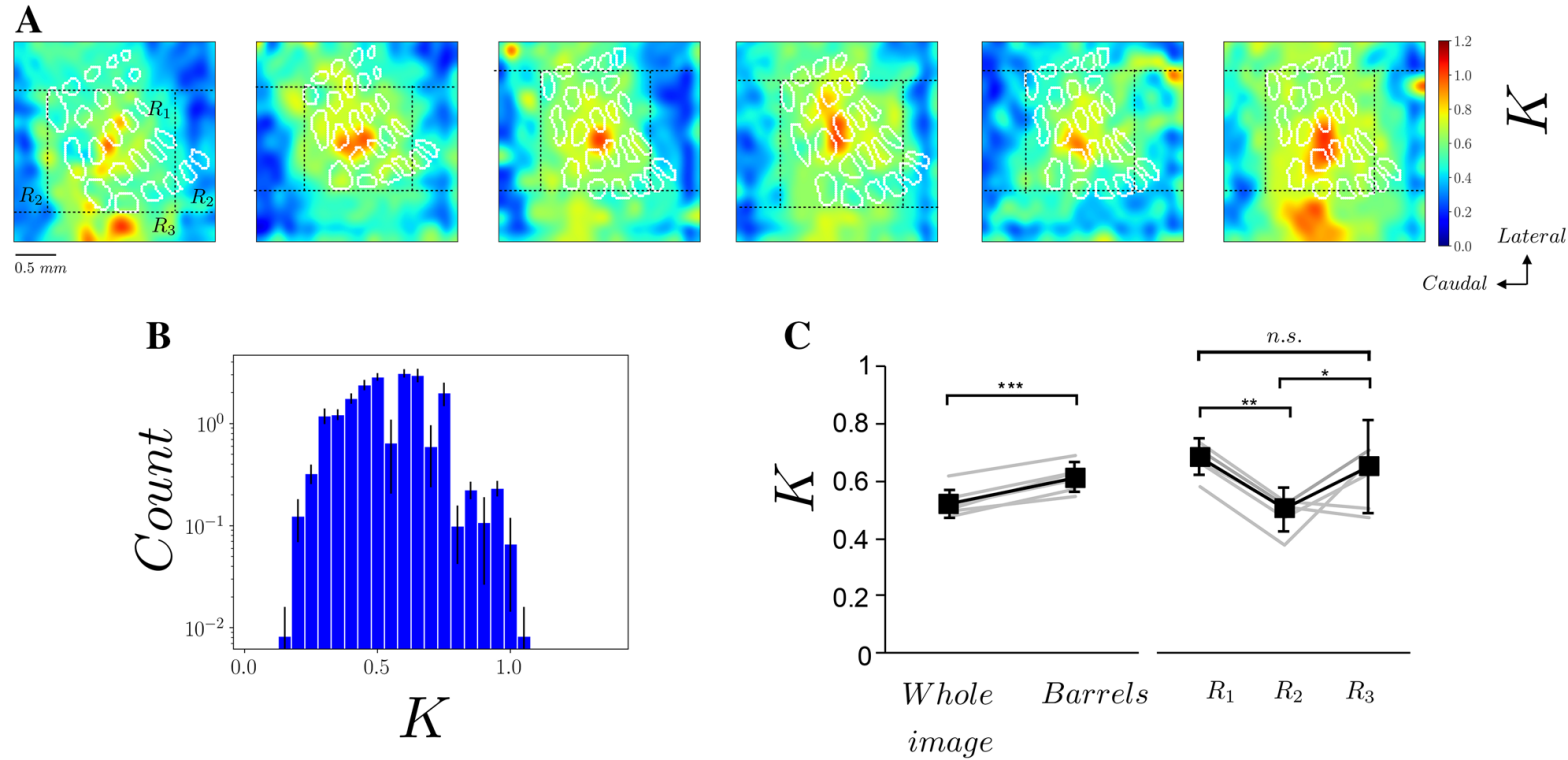
Couplings reconstructed from experimental VSD imaging data. **(A)** The reconstructed spatial distribution of *K* for 6 different mice. The dashed lines are estimated based on the spatial location of histologically reconstructed barrels for each mouse. Horizontal dashed lines correspond to the lateral border of the α barrel and medial border of the δ barrel, respectively. Vertical dashed lines correspond to the caudal border of the α barrel and rostral border of the D4 barrel, respectively. Three regions R_1,2,3_ are defined from these landmarks. Notice that, as explained in the main text, our inversion procedure does not take into account spatial locations higher than the A4 whisker representation, where the activity appears clearly influenced by S2. (**B)** Histogram of the values of *K* reconstructed with the inversion method. The values are mean (+ /− standard deviation) over the 6 mice. (**D)** Mean *K* values computed over 5 different regions for the 6 mice (individual values in grey, mean + /− standard deviation over the 6 animals in black). Whole image stands for the mean over the whole field of view, *Barrels* stands for the mean computed over the pixels located inside the delimited barrels, *** P < 0.001, (paired t-test). *R*_*1,2,3*_ stands for the mean over the region R_1,2,3_ as shown in A, ** P < 0.01, * P < 0.05, n.s. non significan (one way repeated measures analysis of variance followed by an all pairwise multiple comparison procedure (Holm–Sidak method)).

Once validated the procedure on synthetic VSD data, we apply the same procedure to the experimental signal (data illustrated in Fig. 1). In real data we notice that a wave of activity propagates from the top of the image toward the center at time t = 26 ms (Fig. 4A). This wave is probably originating from S2, as suggested in ^20^ (See Fig. 1D). As our model does not account for the interaction between S1 and S2, we consider the spatio-temporal signal up to time t = 28 ms and we do not take into account the spatial activity on the top of the image. The inversion procedure converges to a reasonably good match between the spatio-temporal pattern observed experimentally and the one predicted by the model (see Fig. 4A,B). This can be better appreciated by looking at the time course of VSD activity at different locations. In Fig. 4C we observe a quite good match between the reconstructed and the experimental quantities. Notice that with the initial guess (i.e. a flat $K(x,y)$), we get the uniform pattern of Fig. 2D. The method is thus able to successfully reproduce the anisotropy of the propagating wave. Nevertheless, we cannot reproduce the high activity observed at the top of the image (see Fig. 4A time = 26 ms), which may be due to an interaction with S2. By performing the inference procedure over the 6 mice, we recovered a heterogeneous and non-symmetric distribution of couplings $K(x,y)$ (Fig. 5A). This implies a non-uniform distribution of all the values of $K$ in space, which is robust across the 6 mice (see Fig. 5B). We have observed that the mean value of $K$ over the whole image is significantly lower than the mean value of $K$ estimated inside all the whiskers representation areas, as determined by histological staining of layer IV barrels (paired t-test, P = 0,0004, Fig. 5C). This indicates that the whiskers representation subfield of S1 is a strongly interconnected network with respect to surrounding areas. We have then compared the *K* values in different regions within our field of view. Because the barrel map in Layer 4 varies slightly in shape or position between individuals, we identified three different regions R_1_, R_2_, and R_3_, based on the specific topology of the whisker subfield for each mouse (see Fig. 5A). If the mean *K* value quantified from a central region of the image covering the barrels (R_1_) appeared to be significantly higher than the one quantified from the lateral regions (R_2_, P = 0.0084), it did not differ significantly from the one quantified from the medial side of the image (R_3_, P = 0.5242). This medial region, which stands out of the whiskers representation also showed a significantly higher *K* than the lateral regions (R_3_ vs R_2_, P = 0.0165, one way repeated measures analysis of variance followed by an all pairwise multiple comparison procedure (Holm–Sidak method)). This indicates that this region, which lies partly on the posterior part of the parietal cortex overlaying the anterior extrastriate region of the primary visual cortex^21^ (see Fig. 1D), is highly connected with the whiskers subfield of S1, as suggested by anterograde tracing experiments^30^.

### Suppressive interaction between propagating waves

**Figure 6:**
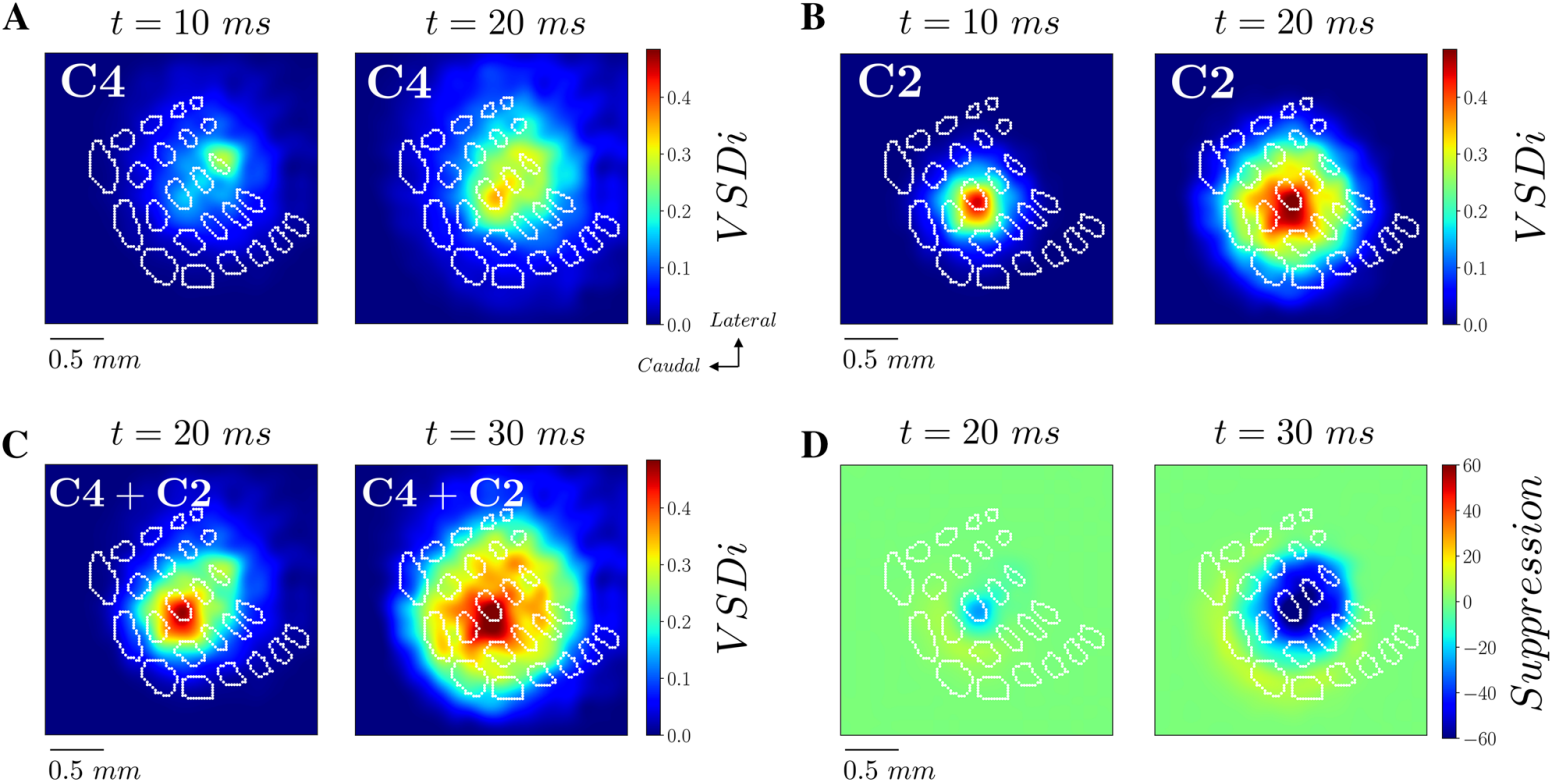
Suppressive interaction between propagating waves in the model. **(A)** VSD activity simulated by stimulation of the C4 whisker at t = 0 ms. (**B)** VSD activity simulated by stimulation of the C2 whisker at t = 0 ms. (**C)** VSD activity simulated by the sequential stimulation of the C4 whisker at t = 0 ms and the C2 whisker at t = 10 ms. (**D)** Difference between data from experiment as in panel (**C)**, and the linear prediction from panel (**A** + **B)** (with proper + 10 ms time shift of data from C2 whisker stimulation**)**, normalized by the maximum of activity in activity of data of panel (**A)**.

**Figure 7:**
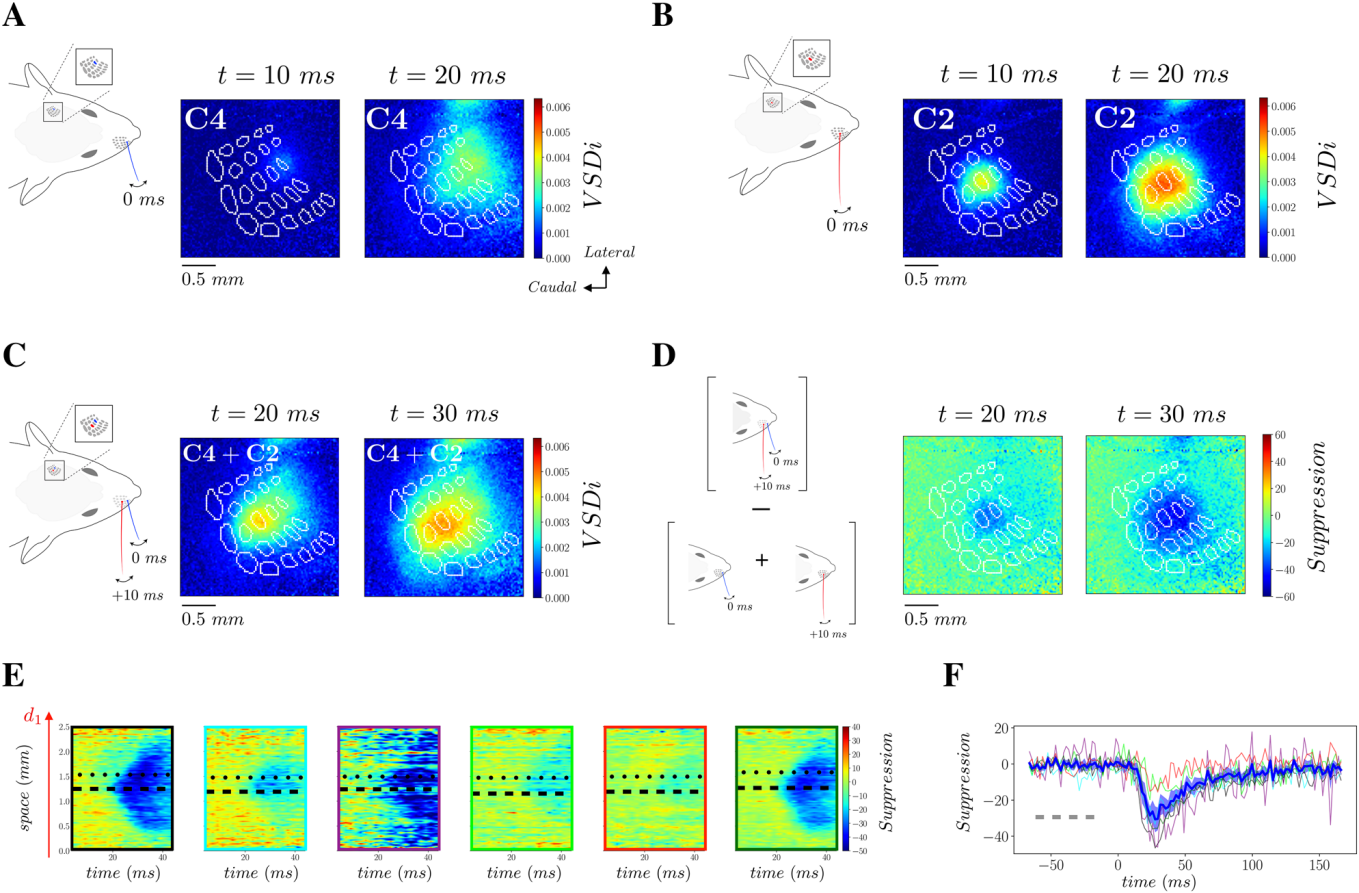
Suppressive interaction between propagating waves. **(A)** VSD activity in response to stimulation of the C4 whisker at time t = 0 ms (**B)** VSD activity in response to stimulation of the C2 whisker at time t = 0 ms. (**C)** VSD activity in response to the sequential stimulation of C4 and C2 whiskers with a delay of 10 ms (**D)** Difference between data from experiment as in panel (**C)**, and the linear prediction from panel (**A** + **B)** (with proper + 10 ms time shift of data from C2 whisker stimulation), normalized by the maximum of activity in data of panel (**A)**. (**E)** Spatio-temporal representation of the suppressive waves for the 6 different mice. Dashed (dotted) black line indicates the location of C2 (C4) barrel. The different color contours stand for different mice. (**F)** Time trace of the suppression at the C2 location (see dashed line in Panel **E**) for the 6 mice (color coded according to the color of the frames in panel **E**). The blue thick line is the mean across mice (the blue shadow indicating the standard deviation).

In previous sections, we have shown that a short stimulus can induce a transition from a lower activity state to a higher activity state that propagates in an anisotropic way in S1 according to the spatially heterogeneous distribution of coupling strengths. The response to an external stimulus delivered at a given spatial location depends on the strength of the input but also on the state of the population of neurons in that specific location. In this network of RS and FS neurons very active states have a lower response to external stimulation, as we have shown in^31^. As a result, each node (which is a network of RS and FS neurons) has a sublinear input–output gain function, at variance with single neurons which have instead a supralinear input–output gain function (the closer to threshold the stronger the response of one neuron). Single node input–output gain function depends on the microscopic organization of RS–FS networks. In a recent work we have shown that a sublinear population response of a network of RS and FS neurons is due to the presence of conductance based interactions (when considering current based interactions an almost linear response was observed)^13,15^. Another crucial factor is a higher gain of inhibitory with respect to excitatory neurons (when considering identical excitatory and inhibitory neurons an almost linear response was also observed)^13,15^. Our single node model contains both these ingredients, i.e. conductance based interactions and a higher gain of inhibitory neurons with respect to excitatory ones. Moreover, in this two dimensional model each node is then connected with the other nodes in space through the connectivity matrix K. By stimulating two different spatial locations, two propagating waves spread from different origins. The activity in each spatial location is a nonlinear (as described sub-linear) function of the inputs received from all the other spatial locations. Hence, we expect this nonlinearity (more precisely a sub-linearity of response in function of the level of activity) to emerge also in this spatially extended model when two propagating waves collide together. In order to verify this hypothesis, we consider the response evoked by the stimulation of two spatially close whisker representations in S1 (C4 and C2, Fig. 6A,B). Each stimulus provokes a propagating wave that spreads in a few milliseconds to the representation of the other whisker. Consequently, when the two stimuli are presented at a short time interval (Fig. 6C), the waves collide together and the representation of one stimulus overlaps the representation of the other. We measured that the activity evoked by the two stimuli presented sequentially is lower than the linear summation of the responses to the two stimuli presented separately. This can be quantified by considering the difference between the actual response and the linear expectation (normalized by the maximum activity to the 1st stimulus response). As we can see in Fig. 6D, this gives rise to a suppressive wave (negative meaning the actual response is lower than the linear expectation) that propagates from the second stimulated whisker’s representation site to the first one. In order to verify this prediction from our simulations, we have applied this stimulation protocol in experiments and measured VSD activity in S1. More specifically, we have first recorded responses to stimuli applied to the C4 and to the C2 whisker independently, and then measured evoked activity when the C2 whisker is stimulated 10 ms after the C4 whisker, by means of VSD imaging. In Fig. 7 we report the results, panel A shows VSD signals imaged in response to a C4 stimulation alone, panel B shows the response to a C2 stimulation alone, and panel C shows the response to the stimulation of the paired stimulation of the C4 and the C2 whiskers with a delay of 10 ms. In panel D we report the suppressive wave as defined for Fig. 6. The suppressive wave was found to be present across the 6 mice even if with different intensities (Fig. 7E–I). This can be clearly seen by looking at the time trace of the suppressive wave in the C2 location that we report in Fig. 7F. All the 6 animals show a peak of suppression, with a mean peak at around -30% with respect to the maximum activity in response to C2 stimulation. Moreover, the strongest suppression arrives close to the C2 barrel-related column (see the location of C2 (dashed line) and of C4 (dotted line) in Fig. 7E). These results show that, as predicted by the model, the activity of the two colliding waves is lower than the linear prediction from the two waves measured separately.

### Employing the inferred model to study complex stimulations

**Figure 8:**
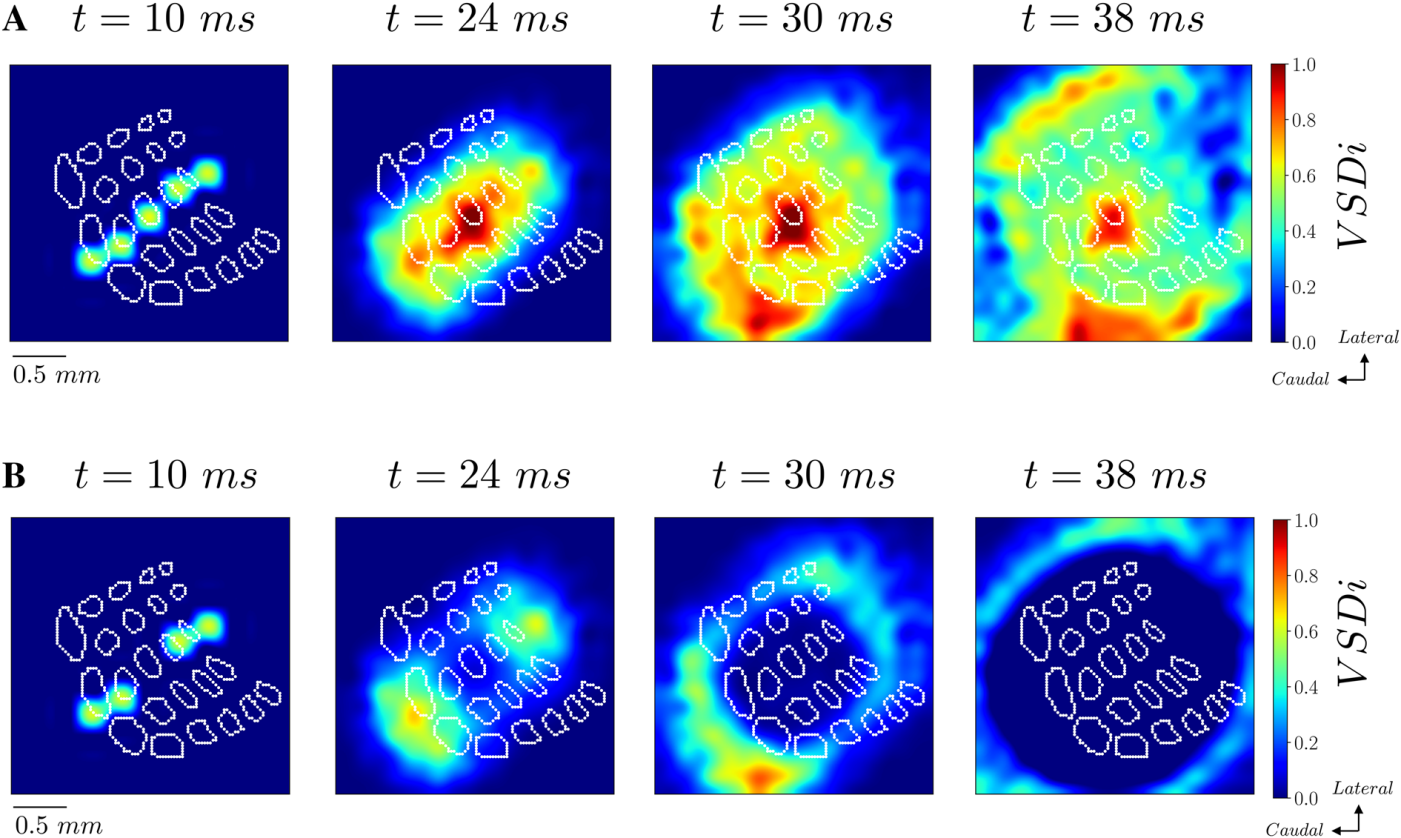
Model prediction of cortical activity evoked by stimulation of the entire C row. **(A)** VSD activity in response to stimulation of the entire C row inferred from the proposed model. (**B)** Difference between VSDi activity in response to the activation of the entire C row (panel **A**) and the activation of only the C2 whisker (Fig. 4A).

The inferred model can be employed to test theoretically the effect of different spatio-temporal whiskers activation. We have studied the effects of the activation of an entire row, more specifically of row C. As show in Fig. 8A, a large propagating wave appears, which strongly resembles the wave of activity that we observed by stimulating only the C2 whisker (see Fig. 4A). Nevertheless, we can highlight differences by subtracting the activity produced by the stimulation of the entire C row minus the activity produced by the stimulation of C2 only. We observe in Fig. 8B that the stimulation of the entire row induces a larger spread of activity in lateral regions of the image, which was only weakly activated by the stimulation of C2. While studying different stimulus combination goes beyond the scope of this work, this result shows that the proposed model can be employed to predict differences in spatio-temporal activity for the desired spatio-temporal whisker stimulation.

## Discussion

In this work, we employed VSD imaging and computational modeling to investigate the origin of anisotropic propagating waves of activity in the mouse somatosensory cortex in response to tactile stimulation. We have developed an inversion method that permits to infer the distribution of spatially distributed cellular parameters in the model directly from experimental VSD recordings. Our results show that a spatially heterogeneous distribution of excitatory synaptic strengths is responsible for the emergence of anisotropic pattern of activity measured in S1. This approach can be extended to a large variety of VSD signal recordings, in different regions and animals, permitting to derive different cellular organization directly from large scale imaging measurements in the brain. Finally, we have employed the model to predict, and then to verify experimentally, a suppressive interaction of propagating waves in S1. This suppression emerges from nonlinearities in the sensitivity to perturbations in networks of excitatory and inhibitory neurons. As we have shown in a recent work^13^, such suppression disappears for current-based interacting neurons or whenever FS and RS neurons have a similar input–output response function (FS cells need to have a higher gain in the input–output function).

It is well known that a fraction of more active neurons in S1, giving rise to a skewed distribution of response probabilities across neurons^32,33^. A recent modeling work ^34^ has shown how a subnetwork of highly connected neurons immersed in a larger network of weakly coupled neurons can give rise to propagating waves in S1 where neurons have a heterogeneous spiking activity. In our model, heterogeneity of neural responses emerges as a direct result of our inversion procedure to match VSD experimental recordings (see the skewed distribution in Fig. 5A). Indeed, a neuron with lower strength of incoming excitatory synapses has a lower activation in response to external stimulus. Moreover, our analysis shows that such heterogeneity has a specific structure in space, responsible for the anisotropic wave observed in VSD recordings. Other sources of heterogeneity in our model could explain a different response of neurons, just like neurons resting potential of membrane time constants. While in this work modifications of excitatory synapses strength were enough to match VSD data in response to whisker stimulations, future works should address how heterogeneity in different parameters can affect the structure of propagating waves.

In this first work we have focused our inference on the spatial distribution of the excitatory coupling between neurons. In principle, the method can be applied to infer different parameters of the model, just like the velocity of propagation v_c_. Here we fitted v_c_ from data (Fig. 1) in order to focus on the couplings but in future works we plan to extend our procedure to infer model parameters without any fitting prior the inference.

Our model limited its analyses to the early response of S1 after tactile stimulation. As already discussed in the result section, a propagating wave originating outside S1 appears around 20 ms after whisker stimulation and propagates toward whiskers representation in S1. This wave is originating in S2 which receives direct tactile sensory inputs from the thalamus^35–37^, as well as a monosynaptic drive from S1 through axons travelling in deep cortical layers ^37^. As shown in ^20^, S2 comprises a discernable topographic arrangement of individual whisker representations, and responds with a similar intensity to individual whisker stimulation as S1, but with a slight delay in time. It may have a key role in the representation of sensory stimuli ^38–42^. An extension of this model to account for S2 may shed light on the cellular organization of the whole somatosensory cortex and the interaction between S1 and S2, directly from VSD measurements.

In this work we have employed a recently developed biologically realistic mean field model of the cortex and arranged it in 2D space to compare with VSD recordings of evoked activity. Our approach employs a low dimensional population model keeping track of fundamental biologically realistic ingredients, such as spike frequency adaptation and conductance based synapses. Nevertheless, a different approach has been recently proposed ^43^, where VSD activity is obtained from numerical simulations of a highly detailed model designed directly from cellular information. While we do not have access to cellular resolution with VSD imaging, the advantage of the approach presented here is to keep the model simple and sufficiently realistic. Indeed, the inversion procedure to infer model parameters from VSD data would be computationally impossible if we were ambitioning to model all cellular details. This method succeeded in calibrating model parameters for an optimal match with VSD imaging data and showed that the anisotropic activity of S1 in response to whisker stimulation can be explained from a spatially heterogeneous coupling between neural populations in S1. On the other side, we have shown that the model is able to encode enough realistic features to correctly reproduce the complex nonlinear effects we have investigated, just like the nonlinear interaction between propagating waves in S1. The computational model designed directly from experimental data paves the way to future studies to investigate the activity in S1 in response to more complex simulations. Taking into account spike frequency adaptation, the model gives the opportunity to make predictions of S1 activity in different brain states. In a recent work we have shown that spike frequency adaptation can model neuromodulation, thus explaining differences in cortical dynamics during awake states, sleep and anesthesia ^44^. The model here developed will permit to study differences in cortical information processing across brain states.

A sublinear interaction of multiple coincident inputs was already found in the somatosensory cortex^45^. In this work authors showed that the earliest cortical response was linear, and the suppression arrived with a finite time delay. We believe that the underlying mechanisms for such a non-linearity at specific spatial locations may be the same as those we find here and that our model may be employed in the future to predict the time course of nonlinearities. At the same time, here we observed that such a nonlinearity propagates in space and in time as a wave. A nonlinear, suppressive, interaction between propagating waves has been recently reported in the visual cortex of the awake monkey in response to two consecutive and spatially closed visual stimuli^13^. We have shown here that this phenomenon appears in a similar protocol also in the somatosensory cortex. The mean field model predicts that this sub-linearity is due to a lower sensitivity to external stimulation of populations of excitatory and inhibitory neurons during high activity states. This prediction can be checked by measuring both the intensity of the suppression and the response of S1 to noisy stimulation, estimating their correlation across different animals and brain states. Moreover the observed suppressive wave, given its robustness across species and brain regions (monkey V1 and mouse S1), is likely to be a general cortical mechanism to discriminate ambiguous stimuli, such as the consecutive stimulation of one whisker followed by the stimulation of another whisker with a time delay Δt (here we used two close whiskers, C2 and C4, and Δt = 10 ms). Future works should investigate the intensity of the suppressive wave by increasing spatial and temporal distance Δt between the two stimulations, in order to further explore the role of suppressive wave to disambiguate external stimulation.

## Materials and methods

### Experimental procedure

Experiments were performed in accordance with the French and European (2010/63/UE) legislations relative to the protection of animals used for experimental and other scientific purposes. Experimental procedures were approved by the local institutional ethical committee registered at the French National Committee of Ethical Reflection on Animal Experimentation under the number #59 (Comité d’éthique en matière d’expérimentation animale Paris Centre et Sud,authorization number: APAFIS#3561-2016010716016314). The study was carried out in compliance with the ARRIVE guidelines.

VSD imaging was performed on six 27–33 days-old C57BL6J mice under isoflurane (induction 3–4%, maintenance 1–1.5%) anesthesia. Paw withdrawal, whisker movement and eye-blink reflexes were suppressed by the anesthesia. A heating blanket maintained the rectally measured body temperature at 37 °C. The respiration of the mice was monitored with a piezoelectric device and the brain state monitored by using two epidural electrodes above the barrel cortex and the frontal cortex ipsilateral to the stimulated whiskers. A metallic fixation post was implanted on the occipital bone with cyanoacrylate glue and dental cement. A ~ 3 × 3 mm craniotomy was made to expose S1. Extreme care was taken at all times not to damage the cortex, especially during the removal of the dura. The voltage-sensitive dye RH1691 (Optical Imaging Ltd, Israel), dissolved at 1 mg/ml in Ringer’s solution containing (in mM): 135 NaCl, 5 KCl, 5 HEPES, 1.8 CaCl2, 1 MgCl2, was topically applied to the exposed cortex and allowed to diffuse into the cortex over 1 h. After removal of the unbound dye, the cortex was covered with agarose (0.5–1% in Ringer’s) and a coverslip.

Cortical imaging was performed through a tandem-lens fluorescence microscope (SciMedia Ldt, USA), equipped with a couple of Leica PlanApo objectives, a 100 W halogen lamp gated with an electronic shutter, a 630 nm excitation filter, a 650 nm dichroic mirror, and a long-pass 665 nm emission filter. We set the field of view to 2.5 × 2.5 mm by using a 5 × objective on the cortex side, and a 1 × objective on the camera side. Images were acquired with a high-speed MiCam Ultima camera (SciMedia Ltd., USA) at 500 Hz. The illumination of the cortical surface started 500 ms before each image acquisition to avoid acquiring signal in the steeper phase of the fluorescence bleaching. Recordings were then of 1 s duration, with 200 ms baseline and 800 ms post stimulation. Variations of the fluorescence signals were initially recorded as variations over the resting light intensity (first acquired frame).

Individual deflections of the right C2, C4 whisker, or paired deflection of the C4 followed by the C2 whisker (with a 10 ms delay) were performed using a multi-whisker stimulator (Jacob et al., 2010) at 0.1 Hz within pseudo randomized sequences containing blank trials (each stimulation being repeated 40 times). Whiskers on the right side were cut to a length of 10 mm and inserted, while keeping their natural angle, in 27G stainless steel tubes attached to piezoelectric benders (Noliac, Denmark), leaving 2 mm between the tip of the tube and the whisker base. Each whisker deflection consisted of a caudal 95 μm displacement (measured at the tip of the tube), a 2 ms rising time, a 2 ms plateau and a 2 ms fall. Specific filters were applied to the voltage commands to prevent mechanical ringing of the stimulators.

Following the experiments mice were perfused with saline followed by paraformaldehyde (4% in 0.1 M phosphate buffer). After an overnight post-fixation in paraformaldehyde, the brains were cut in 100 mm-thick tangential sections that were stained for cytochrome oxidase. Microphotographs of the tangential sections were registered and the barrel maps reconstructed using a method implemented in MATLAB (MathWorks, USA), as previously described ^19^. The functional VSD data were aligned with the reconstructed barrel maps by using the superficial blood vessels as anatomical landmarks.

Acquisition and data preprocessing were done using in-house software (Elphy, G. Sadoc, UNIC-CNRS). Subtraction of a pixel by pixel best fit double-exponential from the averaged unstimulated sequence was used to correct for photobleaching.

## Computational model

We consider a two dimensional square lattice. Every node of the lattice represents the network activity of a large population of excitatory Regular Spiking (RS) neurons and inhibitory (FS) fast spiking neurons (Fig. 1b,c).

### Network model

We consider Adaptive Exponential integrate and fire neurons evolving according to the following differential equations:

1 $${c}_{m}\frac{d{v}_{i}}{dt}={g}_{L}\left({E}_{L}-{v}_{i}\right)+{g}_{L}\Delta {e}^{\frac{{v}_{i}-{v}_{t}}{\Delta }}-{w}_{i}+{I}_{syn},$$

2 $$\frac{d{w}_{i}}{dt}=-\frac{{w}_{i}}{{\tau }_{w}}+b{\sum }_{{t}_{sp}\left(i\right)}\left(t-{t}_{sp}\left(k\right)\right),$$

where *c*_*m*_ = 100 pF is the membrane capacity, *v*_*i*_ is the voltage of neuron *i* and, whenever *v*_*i*_ > v_th_ = −50 mV at times t_sp_(k), *v*_*i*_ is reset to its resting value *v*_*rest*_ = −65 mV. The leak term has a conductance *g*_*L*_ = 10nS and a reversal *E*_*L*_ = −65 mV. The exponential term has a different strength for regular-spiking (RS) and fast-spiking (FS) cells, i.e. $\Delta =2$ mV ($\Delta =0.5$ mV) for excitatory (inhibitory) cells. The variable *w*_*i*_ mimicks the dynamics of spike frequency adaptation. Inhibitory neurons are modeled according to physiological insights as the FS neurons with no adaptation, while the excitatory RS neurons have a lower level of excitability due to the presence of adaptation. Here we consider $b=60$ pA and ${\tau }_{w}=500$ ms, if not stated otherwise. The synaptic current impinging on the postsynaptic neurons $k$, ${I}_{syn}$, is modeled as:

3 $${I}_{syn}=\left({E}_{e}-{v}_{k}\right){G}_{syn}^{e}+\left({E}_{i}-{v}_{k}\right){G}_{syn}^{i},$$

4 $${G}_{syn}^{\left(e,i\right)}\left(t\right)={Q}_{\left(e,i\right)}{\sum }_{n}\Theta \left(t-{t}_{sp}\left(n\right)\right){e}^{\frac{t-{t}_{sp}\left(n\right)}{\tau }},$$

where ${Q}_{e}$ (${Q}_{i}$) is the excitatory (inhibitory) quantal conductance. The variable $\tau =5$ ms is the decay timescale of excitatory and inhibitory synapses, and $\Theta$ is the Heaviside step function. The summation runs over the over all the pre-synaptic spiking times ${t}_{sp}(n)$. We set ${Q}_{e}=1.5$ nS and ${Q}_{i}=5$ nS. We then consider a random network with p = 5% of connectivity and 80% of excitatory neurons. The parameters are chosen according to biological realism for which this model gives rise to asynchronous irregular activity states as observed in the primate visual cortex ^15^.

### Spatially extended two dimensional mean field model

The activity of the network is simulated using a mean field model, capable to predict spontaneous activity in both asynchronous and bistable UP–DOWN states dynamics ^15^. Connecting several mean field models in space, we obtain the following equations for the spatially extended lattice model:

5 $$T\frac{\partial {r}_{E}(x,y,t)}{\partial t}=-{r}_{E}\left(x,y,t\right)+{F}^{E}\left({r}_{E}^{inp}\left(x,y,t\right),{r}_{I}^{inp}\left(x,y,t\right),w\left(x,y,t\right)\right)$$

6 $$T\frac{\partial {r}_{I}(x,y,t)}{\partial t}=-{r}_{I}\left(x,y,t\right)+{F}^{I}\left({r}_{E}^{inp}\left(x,y,t\right),{r}_{I}^{inp}\left(x,y,t\right)\right)$$

7 $${\tau }_{w}\frac{\partial w(x,y,t)}{\partial t}=-w\left(x,y,t\right)+b{r}_{E}\left(x,y,t\right),$$

where r_E/I_(x,y) represents the instantaneous firing rate of excitatory/inhibitory neurons in the location (x,y) and F^E/I^ is the transfer function of excitatory/inhibitory neurons. The variables ${r}_{E/I}^{inp}$ are the net input received by node (x,y) from the rest of the lattice, which can be written as:

8 $${r}_{E}^{inp}\left(x,y,t\right)=\int d{x}_{1}\int d{y}_{1}{r}_{E}\left({x}_{1},{y}_{1},t-\frac{d}{{v}_{c}}\right){G}^{E}\left(x,y;{x}_{1},{y}_{1}\right)$$

9 $${r}_{I}^{inp}\left(x,y,t\right)=\int d{x}_{1}\int d{y}_{1}{r}_{I}\left({x}_{1},{y}_{1},t-\frac{d}{{v}_{c}}\right){G}^{I}\left(x,y;{x}_{1},{y}_{1}\right),$$

where *d* is the distance between (x,y) and (x_1_,y_1_), ${d}^{2}=(x-{x}_{1}{)}^{2}+(y-{y}_{1}{)}^{2}$ and ${G}^{E}(x,y;{x}_{1},{y}_{1})$ is the effective excitatory coupling between the node (x,y) and the node (x_1_,y_1_). The parameter v_c_ = 0.1 mm/ms is the axonal conduction speed, and T = 5 ms is the decay time of population rate. The functions F^E,I^ are the transfer functions of excitatory/inhibitory neurons and are calculated according to a semi-analytical method ^12,15^ through an expansion in function of the three statistics of neurons voltage, i.e. its average ${\mu }_{V}$ , its standard deviation ${\sigma }_{V}$ and its autocorrelation time ${\tau }_{V}$:

10 $${F}_{\nu }=\frac{1}{2{\tau }_{V}}erfc\left(\frac{{V}_{thre}^{eff}-{\mu }_{V}}{\sqrt{2}{\sigma }_{V}}\right),$$

where *erfc* is the Gauss error function, ${V}_{thre}^{eff}$ is an *effective* or *phenomenological threshold.* This threshold is expressed as a first order expansion with some fitting coefficients in function of $({\mu }_{V},{\sigma }_{V},{\tau }_{V})$, which are calculated from shot-noise theory ^46^. Introducing the following quantities:

11 $${\mu }_{Ge}\left({r}_{E},{r}_{I}\right)={r}_{E}{K}_{E}{\tau }_{E}{Q}_{E }$$

12 $${\sigma }_{Ge}\left({r}_{E},{r}_{I}\right)=\sqrt{\frac{{r}_{E}{K}_{I}{\tau }_{e}}{2}}{Q}_{E}$$

13 $${\mu }_{Gi}\left({r}_{E},{r}_{I}\right)={r}_{I}{K}_{I}{\tau }_{I}{Q}_{I}$$

14 $${\sigma }_{Gi}\left({r}_{E},{r}_{I}\right)=\sqrt{\frac{{r}_{I}{K}_{I}{\tau }_{i}}{2}}{Q}_{I},$$

where K_E/I_ is the amount of incoming synapses related to pre-synaptic excitatory/inhibitory neurons (we consider a network of N = 10,000 neurons inside each node of the ring), we obtain the following equations for the voltage moments:

15 $${\mu }_{V}\left({r}_{E},{r}_{I},w\right)=\frac{{\mu }_{Ge}{E}_{e}+{\mu }_{Gi}{E}_{i}+{g}_{L}{E}_{L}-w}{{\mu }_{G}}$$

16 $${\sigma }_{V}\left({r}_{E},{r}_{I}\right)=\sqrt{\sum {K}_{s}{r}_{s}\frac{({U}_{s}\cdot {\tau }_{s}{)}^{2}}{2\left({\tau }_{m}^{eff}+{\tau }_{s}\right)}}$$

17 $${\tau }_{V}\left({r}_{E},{r}_{I}\right)=\left(\frac{{\sum K}_{s}({U}_{s}\cdot {\tau }_{s}{)}^{2})}{{\sum (K}_{s}{r}_{s}({U}_{s}\cdot {\tau }_{s}{)}^{2}/({\tau }_{m}^{eff}+{\tau }_{s}))}\right).$$

Details on fitting procedure and the comparison between mean field predictions and network simulations are reported in ^15^.

### Spatial coupling

The function G(x,y,x_1_,y_1_) represents the effective directional and anisotropic coupling from site (x_1_,y_1_) to site (x,y). Notice that, in a mean field description as the present one, this coupling can be interpreted also as the average amount of incoming connection that a neuron in (x,y) receives from a neuron in (x_1_,y_1_). We assume this coupling to be decreasing with the distance *d* between (x_1_,y_1_) and (x,y), and proportional to the average amount of incoming inputs received by a neuron in (x,y), K(x,y):

18 $$G\left(x,y;{x}_{1},{y}_{1}\right)=K\left(x,y\right){e}^{-\frac{d}{\lambda }}$$

where λ is a scale determining the decay and K(x,y) can be interpreted as an effective coupling of (x,y) with the rest of the lattice. In this work we have fixed λ = 0.8 mm (notice that the lattice we consider has an edge L = 2.5 mm). We have also verified that small modification of the value of λ did not affect the results of our analyses.

### Inversion procedure

We search for the coupling function K(x,y), such that the spatio-temporal pattern measured experimentally matches the one predicted by the model. We consider an experimental setup where a propagating wave is activated in C4. Accordingly, all neurons in C4 receive a fast and spatially local external excitatory input that activates the propagating wave. The input is an excitatory poissonian train of spikes of duration T_inp_ = 2 ms and amplitude A_inp_ = 10 Hz. We then consider a coarse grained version of the spatial model with M = 20 pixels in each direction. The function K(x,y) is then a MXM matrix. For a specific matrix K we simulate the model and obtain a time varying 2D matrix VSDi_model_ to be compared with VSDi_exp_. We introduce the distance D such that:

19 $$D = D\left( {{\text{~}}VSD{I_{model}},{\text{~}}VSD{I_{exp}}} \right) = \smallint dx\smallint dy\smallint dt\frac{{\left| {VSD{I_{exp}} - VSD{I_{model}}} \right|}}{{\left| {VSD{I_{exp}}} \right|}}$$

**Figure 9:**
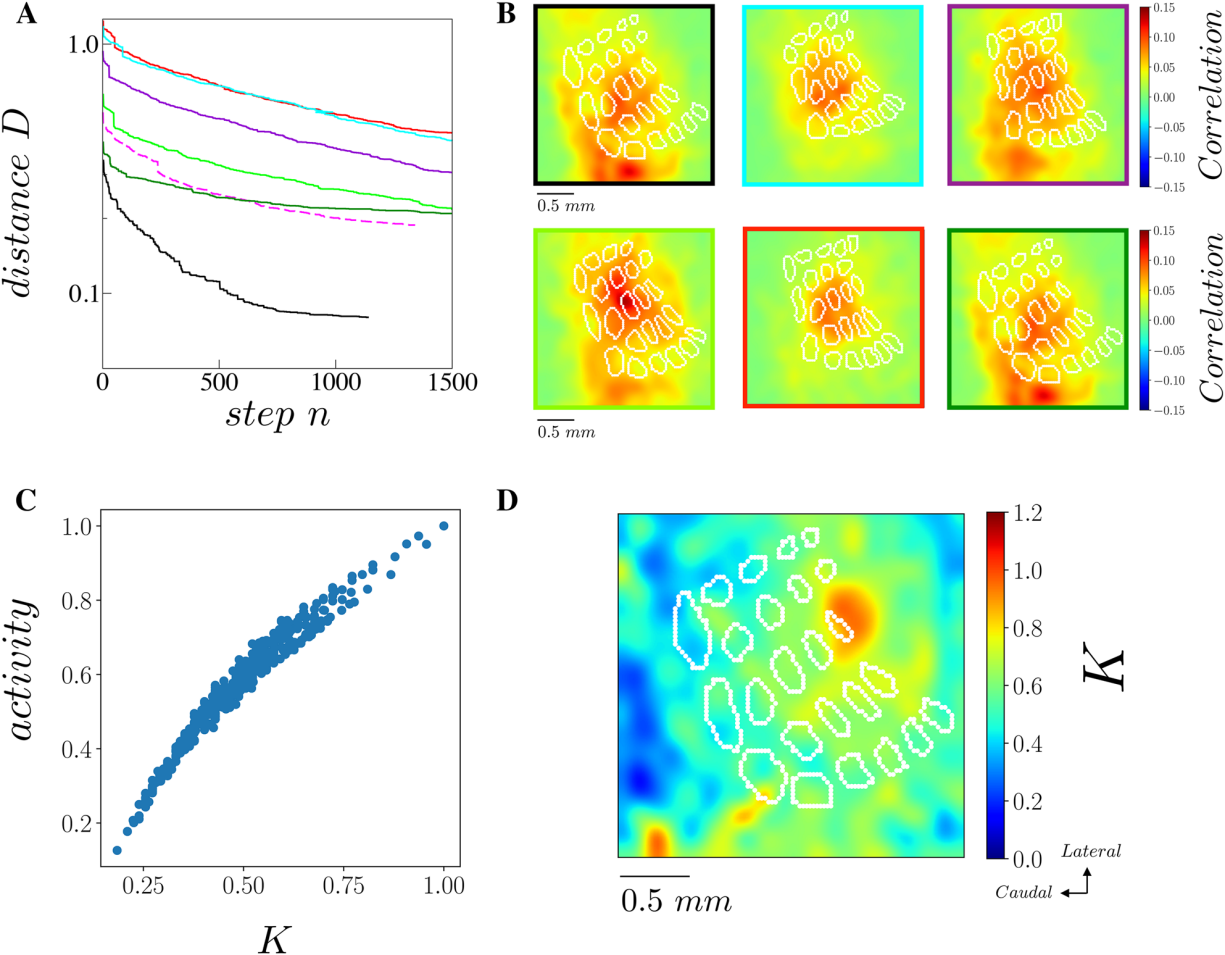
Inversion procedure. **(A)** Distance D (from Eq. 19 in function of the step number *n* of the inversion procedure. Solid lines show the convergence for the 6 different mice (black line is from the mouse illustrated in Figs. 1,4,7A) and the dashed line is obtained from the inversion procedure applied to VSDi obtained by stimulating C4 (same mouse as black line). (**B)** Correlation (averaged over 20 ms) between reconstructed and experimental VSDi for the 6 mice. The color of the frame is indicative of the identity of the mouse as in panel **(A)**. (**C)** Each dot shows the maximum activity (in time) and the value of *K* at each pixel (Pearson correlation coefficient p_c_ = 0.95). (**D)** The reconstructed spatial distribution of *K* from the inversion procedure applied to recorded VSDi signals in response to C4 whisker stimulation.

The inversion procedure works as follows: we start with an homogeneous matrix K_0_(x,y) as initial conditions and, at each step n = 1..N of the procedure, we pick randomly a location (x_c_, y_c_) and increase or decrease the value of the matrix of a quantity ε. We thus obtain the matrix K_n_(x,y) such that K_n_(x_c_, y_c_) = K_n−1_(x_c_, y_c_) + ε. We then simulate the 2D mean field model with K_n_ obtaining VSDi_model_(n) and the new distance D_n_ = D( VSDi_model_(n), VSDi_exp_(n)). If the distance d_n_ < d_n−1_ we accept the modification to K, if not we set K_n_ = K_n−1_. The procedure continues up to when we reach a satisfactory (smaller then 1) distance d_N_. In the inversion procedure reported here we have chosen ε = 0.1, and employed N around 5000 up to a distance of D_N_ arounds 0.1. The time window in which we perform the inversion procedure is Δt = 20 ms. In Fig. 9A we report the distance D_n_ in function of n in all the inference procedures we performed (across the 6 mice). We see a slow but steady decay of D_n_ implying that the procedure indeed is converging to the best solution. In Fig. 9B we report instead correlation between the inferred activity and the experimental activity (across the 6 mice). We observe that we have a higher correlation in the whisker representation, where the VSD activity is stronger. The correlation is positive in all spatial locations, indicating a good agreement between data and inferred model. Moreover, we observed that higher values in *K* appear in the direction of VSD spread.

In Fig. 9C is plotted the maximum activity as a function of the *K* value in all the spatial locations, which appeared almost linearly correlated (Pearson correlation coefficient p_c_ = 0.95). On the other side, the values of *K* are not correlated to the distance from the evoked whisker, as demonstrated by the average values of *K* in regions R_2_ and R_3_ (see Fig. 5). In Fig. 9C is plotted the maximum activity as a function of the K value in all the spatial locations, which appeared almost linearly correlated (Pearson correlation coefficient pc = 0.95). On the other side, the values of K are not correlated to the distance from the evoked whisker, as demonstrated by the average values of K in regions R2 and R3 (see Fig. 5). Finally, we performed the inversion procedure by stimulating C4 (see Fig. 9D). We found that the *K* value is stronger in C4, the stimulus location in this case. This analysis shows that the inferred values of *K* in the exact location of the stimulus are affected by a bias and depend on the strength of the stimulation. Notice that, nevertheless, our statistical results (e.g. a stronger coupling in the region of barrels representation, see Fig. 5C) stay valid, as well as the presence of the suppressive wave (see Fig. 7E).
